# The Association between Temporomandibular Disorder and Sleep Apnea—A Nationwide Population-Based Cohort Study

**DOI:** 10.3390/ijerph17176311

**Published:** 2020-08-30

**Authors:** Ju-Hui Wu, Kun-Tsung Lee, Chia-Yu Kuo, Chih-Hung Cheng, Jih-Yu Chiu, Jen-Yu Hung, Chung-Yao Hsu, Ming-Ju Tsai

**Affiliations:** 1 Department of Dentistry, Kaohsiung Medical University Hospital, Kaohsiung Medical University, Kaohsiung 807, Taiwan; wujuhui1020@gmail.com (J.-H.W.); denzellee63@gmail.com (K.-T.L.); asohowto@gmail.com (J.-Y.C.); 2 Department of Oral Hygiene, College of Dental Medicine, Kaohsiung Medical University, Kaohsiung 807, Taiwan; 3 Division of Pulmonary and Critical Care Medicine, Department of Internal Medicine, Kaohsiung Medical University Hospital, Kaohsiung Medical University, Kaohsiung 807, Taiwan; goba2356@gmail.com (C.-Y.K.); markbruse617@gmail.com (C.-H.C.); jenyuhung@gmail.com (J.-Y.H.); 4 Sleep Disorders Center, Kaohsiung Medical University Hospital, Kaohsiung Medical University, Kaohsiung 807, Taiwan; cyhsu@kmu.edu.tw; 5 School of Medicine, College of Medicine, Kaohsiung Medical University, Kaohsiung 807, Taiwan; 6 Department of Respiratory Care, College of Medicine, Kaohsiung Medical University, Kaohsiung 807, Taiwan; 7 Graduate Institute of Clinical Medicine, College of Medicine, Kaohsiung Medical University, Kaohsiung 807, Taiwan; 8 Department of Neurology, Kaohsiung Medical University Hospital, Kaohsiung Medical University, Kaohsiung 807, Taiwan

**Keywords:** temporomandibular disorder, sleep apnea, sleep disordered breathing

## Abstract

An increased incidence of temporomandibular disorders (TMD) among patients with sleep apnea (SA) has been reported. However, the association between TMD and SA has not been demonstrated in a large-scale study. This population-based cohort study with the Taiwan National Health Insurance (NHI) Research Database aimed to understand the association between SA and TMD. We identified adult patients with suspected SA (identified with diagnostic codes) and excluded those diagnosed with TMD prior to SA. Patients with SA diagnosis after polysomnography were also identified as probable SA patients. The index dates were the dates of their initial SA diagnosis. Ten control subjects were matched, by age and sex, to each SA patient, and were assigned the same index dates as the SA patients. In total, 10,408 suspected SA patients (including 4105 probable SA patients) matched to 104,080 control subjects (including 41,050 subjects matched to the probable SA patients) in this study. The TMD incidence rate was significantly higher in the SA patients than in the control subjects (2.8 vs. 1.0 per thousand-patient-year in probable SA patients vs. the corresponding control subjects, with an adjusted incidence rate ratio [95% confidence interval] = 2.5 [2.3–2.7], *p* < 0.0001). SA patients significantly showed a higher cumulative incidence of TMD than the corresponding control subjects (*p* < 0.0001). Multivariable Cox regression analysis revealed SA as an independent risk factor for the development of TMD (adjusted hazard ratio = 2.5 [1.7–3.7], *p* < 0.0001). In summary, this study confirmed an increased TMD incidence in the SA patients. While treating TMD patients, dentists should pay careful attention to the potential underlying SA.

## 1. Introduction

Temporomandibular disorders (TMDs) are musculoskeletal disorders characterized by persistent pain in the temporomandibular joint, masticatory muscles, and periauricular region [1]. Based on clinical evaluation, the prevalence of TMD was 6–93% in the general population [2]. It has been considered a complex and multifaceted disease process [3]. Biomechanical, neuromuscular, neurobiological, and biopsychosocial factors may contribute to the disorder [4]. Risk factors contributing to TMD includes age, genetic factors, sex, stress, anxiety, occlusion, poor posture, rheumatoid arthritis, and dysfunctional breathing [2,5,6]. TMD develops at a markedly higher rate in individuals with relatively poorer health status, whether in the form of other pain conditions, comorbid diseases, poor sleep quality, or cigarette smoking [7].

Being the most common type of sleep-disordered breathing, sleep apnea (SA) is usually characterized by repetitive shallowing and stopping of breaths during sleep, which is usually accompanied with intermittent hypoxia, arousal, and sleep fragmentation [8]. Polysomnography (PSG) is usually performed to establish the diagnosis of SA [9]. Most (>90%) cases are obstructive sleep apnea (OSA), while a small proportion of cases are central sleep apnea (CSA) [10,11]. SA has been associated with a variety of diseases, such as arrhythmia, hypertension, ischemic heart disease, stroke, metabolic syndrome, diabetes mellitus, chronic kidney disease, erectile dysfunction, hormonal dysfunction, and dementia [8,12,13,14].

Several previous studies have shown the association between sleep disorders and TMD [6,7,15], while only few of them discussed the association between SA and TMD. In the OPPERA (Orofacial Pain: Prospective Evaluation and Risk Assessment) cohort, which recruited subjects from 2006 to 2008 and followed them through 2012, signs/symptoms of OSA were associated with higher risk of incident TMD [6]. Because both SA and TMD are associated with several comorbidities, the association between SA and TMD might be very complex. A large-scale cohort study that takes comorbidities into the analyses is therefore needed. This large population-based cohort study aimed to confirm the association between SA and TMD by demonstrating a higher TMD incidence in SA patients.

## 2. Materials and Methods 

### 2.1. Study Design

This study is a retrospective cohort study using a large nationwide population-based database.

### 2.2. Data Sources

Since March 1995, the Taiwan National Health Insurance (NHI) has covered outpatient care, dental services, hospital inpatient care, and drug prescriptions. In the population of about 23 million people, the coverage rate reached 99% in 2004 [8,16]. From the NHI Research Database (NHIRD) managed by the National Health Research Institutes in Taiwan, we obtained the Longitudinal Health Insurance Database 2005 (LHID2005), a cohort of one million randomly sampled subjects in the NHI system in 2005, for this study. For confidentiality, patient identification was encrypted in the database.

### 2.3. Ethical Consideration

The Institutional Review Board of Kaohsiung Medical University Hospital approved this study (KMUH-IRB-EXEMPT-20130034).

### 2.4. Sleep Apnea Cohorts

**Figure 1:**
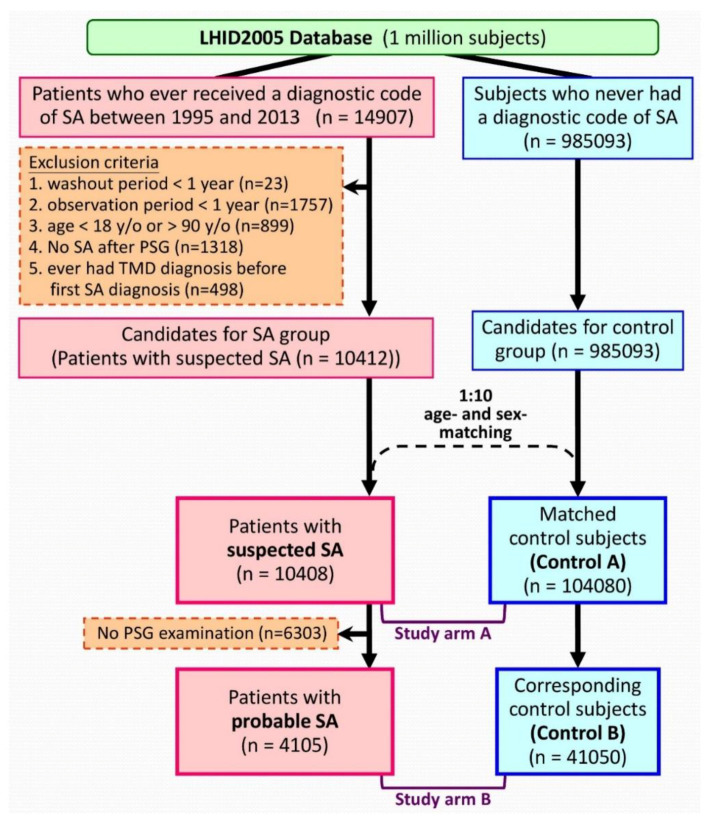
Algorithm to identify the study population. Abbreviations: LHID2005 = Longitudinal Health Insurance Database 2005; SA = sleep apnea; TMD = temporomandibular disorder; PSG = polysomnography; y/o = years old.

Patients with “suspected SA” were identified using an algorithm (Figure 1). Patients that received a diagnostic code of SA between 1995/03/01 and 2013/12/31 were identified initially. The International Classification of Diseases, 9th Revision, Clinical Modification (ICD-9-CM) codes of 780.51, 780.53, and 780.57 were used for the diagnosis of SA [8,17]. The ICD-9-CM codes of 524.6 was used to identify the diagnosis of TMD [18]. Each patient was followed from the date of first SA diagnosis (the index date). The exclusion criteria included: patients with insufficient (<1 year) washout periods (between NHI enrollment and the index date); patients with insufficient (<1 year) follow-up period; patients that received a TMD diagnosis before the index date; aged less than 18 or more than 90 years. Therefore, the “suspected SA cohort” consisted of SA patients identified with diagnostic codes from the database.

In the suspected SA patients, those who had retained their SA diagnosis after a PSG examination were further extracted and were defined as the “probable SA cohort.” Theoretically, these “probable SA patients” might have PSG-confirmed SA.

### 2.5. Control Cohorts

We randomly selected 10 age- and sex-matched control subjects for each suspected SA patient. The index dates of control subjects were assigned based on those of their corresponding SA patients. We applied the same exclusion criteria while selecting the control subjects in the matching process.

### 2.6. Definitions of Variables

The development of TMD was taken as the endpoint in the current study. Only those whose TMD diagnosis appeared at least three times in outpatient claims or at least one time in inpatient claims were considered TMD patients, to increase the accuracy of the diagnosis. A comorbidity was identified by the first appearance of its corresponding diagnostic codes (before the index date), and was confirmed by the appearance in at least three outpatient claims or an inpatient claim. We calculated the Charlson Comorbidity Index (CCI) score of each subject [19].

Each subject was followed from the index date to either the development of TMD, termination of the record due to death or withdrawal from NHI, or the end of 2013 (the end of study period), whichever came first.

### 2.7. Statistical Analysis

The current study comprised two study arms with the same statistical analyses: study arm A comparing suspected SA cohort and control A cohort; study arm B comparing probable SA cohort and control B cohort. Firstly, the demographic information was compared between SA cohorts and the corresponding control cohorts with Student’s t-test and Pearson χ^2^ test for continuous and categorical variables, respectively. The incidence rate of TMD was the number of TMDs occurred in the follow-up period divided by the total person-year. By calculating the incidence rate ratio (IRR), the incidence rates of TMD in the SA cohorts and the corresponding control cohorts were compared. With the assumption that the occurrence of TMD followed a Poisson probability distribution, the 95% confidence interval (CIs) of the IRRs were estimated. We also performed stratified analyses by classifying the subjects based on sex, age, residency, income level, or presence of a comorbidity. The cumulative incidence of TMD was estimated with the Kaplan–Meier method and compared with log-rank test. In order to further assess the effect of SA, multivariable Cox proportional hazards regression analyses, adjusting for sex, age, residency, income level, and comorbidities, were performed. Multivariable Cox regression analyses, built by a backward variable selection method, eliminating variables with a *p*-value of less than 0.05, were used to identify the risk factors for developing TMD in SA patients. The adjusted IRRs and hazard ratios (HRs) were adjusted for sex, age group, residency, income level, and the presence of comorbidities (except for the stratifying variable).

We used the Statistical Analysis System (SAS) system (version 9.4 for Windows, SAS Institute Inc., Cary, NC, USA) for extraction and computation of data, data linkage, processing and sampling, and statistical analysis. We set the statistical significance level at a two-sided *p* value of less than 0.05.

## 3. Results

A total of 10,408 suspected SA patients, including 4105 probable SA patients, were identified and were matched to control subjects (104,080 and 41,050 subjects in the control A and control B cohorts, respectively) by age and sex using the algorithm (Figure 1). Table 1 presents the baseline characteristics of the cohorts. Patients in the SA cohorts had more comorbidities than the subjects in the corresponding control cohorts (Table 1).

The suspected SA patients had a significantly higher TMD incidence rate than the subjects in the control A cohort (2.9 vs. 1.1 per thousand-patient-year, adjusted IRR [95% CI] = 2.0 [1.9–2.1], *p* < 0.0001). A significantly higher TMD incidence rate was also observed in patients with probable SA than in the subjects in the control B cohort (2.8 vs. 1.0 per thousand-patient-year, adjusted IRR [95% CI] = 2.5 [2.3–2.7], *p* < 0.0001) (Table 2). Stratified analyses showed that SA patients had significantly higher TMD incidence rates than the corresponding control subjects in all strata (Table 2).

**Figure 2:**
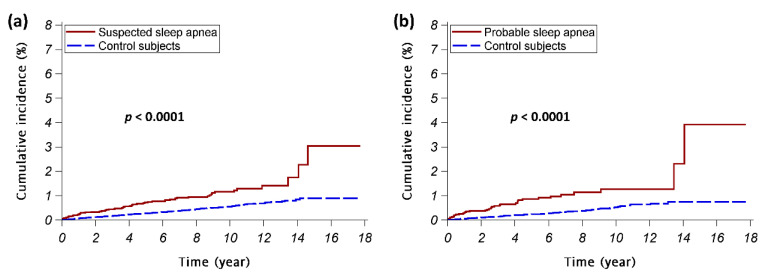
The cumulative incidences of temporomandibular disorder. The red continuous lines show the cumulative incidence of temporomandibular disorder for the sleep apnea patients; the blue dashed lines show the cumulative incidence of temporomandibular disorder for the control subjects. (**a**) Study arm A (the suspected sleep apnea cohort vs. control A cohort); (**b**) Study arm B (the probable sleep apnea cohort vs. control B cohort).

**Figure A1:**
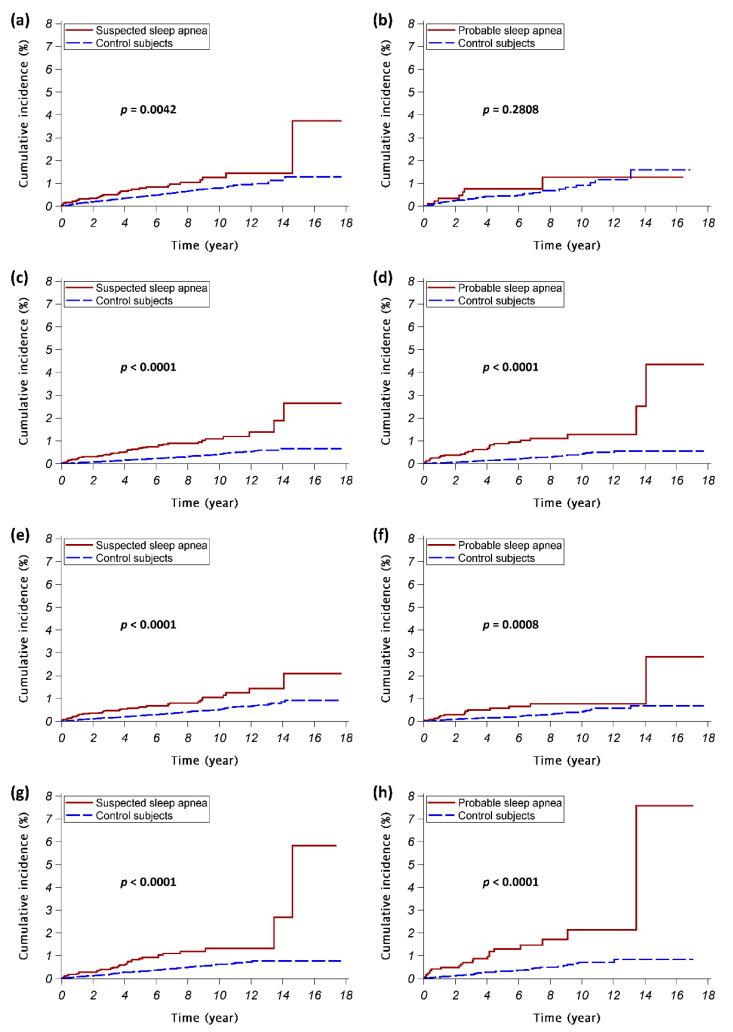
The cumulative incidences of temporomandibular disorder in female subjects (**a**,**b**), male subjects (**c**,**d**), subjects ≤ 50 years of age (**e**,**f**), and subjects > 50 years of age (**g**,**h**). The analyses were taken in study arm A (suspected SA vs. control A) (**a**,**c**,**e**,**g**) and study arm B (probable SA vs. control B) (**b**,**d**,**f**,**h**). The red continuous lines and blue dashed lines show the cumulative incidence of temporomandibular disorder for the sleep apnea (SA) patients and the control subjects, respectively.

The suspected SA cohort had a significantly higher cumulative TMD incidence than the control A cohort (*p* < 0.0001); the probable SA cohort also had a significantly higher cumulative TMD incidence than the control B cohort (*p* < 0.0001) (Figure 2). Consistent results were noted in stratified analyses except for female subjects in study arm B (Figure A1 in Appendix A).

**Figure 3:**
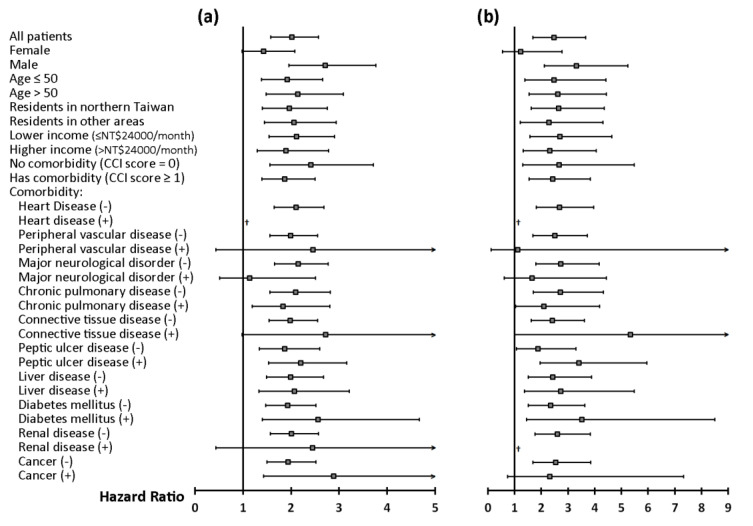
Stratified analyses of multivariable Cox analyses. (**a**) Study arm A (suspected SA vs. control A); (**b**) Study arm B (probable SA vs. control B). Adjusted hazard ratios (95% confidence intervals) of sleep apnea, adjusted for sex, age, residency, income level, and the presence of various comorbidities (except for the stratifying variable) are presented. ^†^ Hazard ratio cannot be estimated because of small sample size. Abbreviations: SA = sleep apnea; CCI = Charlson Comorbidity Index.

Multivariable Cox proportional hazards regression analyses found that SA was an independent risk factor for the development of TMD (adjusted HR [95% CI] = 2.0 [1.6–2.6] and 2.5 [1.7–3.7] in study arm A and B, respectively; both *p* < 0.0001) (Figure 3 and Table A1 in Appendix A). Stratified analyses showed SA as an independent risk factor for incident TMD in nearly all strata (Figure 3).

Using multivariable Cox regression models, we tried to find the risk factors for developing TMD in SA patients. In suspected SA patients, the risk factors for incident TMD included peptic ulcer disease (adjusted HR [95% CI] = 2.0 [1.3–3.2], *p* = 0.0017) and cancer (adjusted HR [95% CI] = 2.4 [1.3–4.4], *p* = 0.0065) (Table 3). In probable SA patients, peptic ulcer disease was found as the only risk factor for incident TMD (adjusted HR [95% CI] = 3.1 [1.6–6.1], *p* = 0.0010).

## 4. Discussion

SA patients had a significantly higher TMD incidence than the control subjects in this large nationwide, population-based cohort study. The analyses in the study arm A, which included SA patients identified by only using the ICD-9-CM codes (i.e., suspected SA cohort), showed similar results as the analyses in the study arm B, which included SA patients diagnosed with SA after PSG (i.e., probable SA cohort). After being adjusted for sex, age, and comorbidities, SA remained an independent risk factor for the development of TMD.

This study is the first and largest long-term, population-based cohort study to survey TMD incidence in SA patients. In line with our finding, several previous studies have shown an association between SA and TMD [20]. Indeed, disturbed sleep may interfere with patients’ daily function, contributing to an increase in their sensitivity to pain [21]. Sleep disturbance may increase pain sensitivity in patients with chronic pain and may create a self-perpetuating cycle of sleep disruption, more pain, and depression. A bidirectional association between OSA and TMD has been suggested, which is evident in their prevalence rates: an increased prevalence of TMD in OSA patients [22] and an increased prevalence of OSA in TMD patients [15]. OSA has been reported as a common comorbidity in general chronic pain populations, with a pooled prevalence of about 37%, as well as in TMD patients, with a frequency of 28.6% [15]. Women with TMD present more respiratory effort-related arousals than healthy controls [23], predisposing them to the development of OSA. 

As this study showed that SA patients had a higher risk of subsequent TMD, it is suggested that physicians treating SA patients should pay careful attention to the possible associated chronic pain, which affects the quality of sleep. Moreover, as the current study revealed that SA was an independent risk factor for the development of TMD, dentists must pay careful attention to the possible underlying SA while treating TMD patients so that appropriate referrals may be made to improve treatment outcomes.

Several mechanisms might be underlying the close relationship between OSA and TMD [24]. Firstly, inadequate and/or disrupted sleep in OSA patients might enhance pain sensitivity, contributing to hyperalgesia, an important feature found in many TMD patients [25]. Secondly, OSA patients usually display chronic intermittent hypoxemia, which increases the levels of inflammatory cytokines, contributing to the pathogenesis of multiple comorbidities [26]. As higher plasma levels of inflammatory cytokines were reported both in OSA and TMD [27,28], we postulated that OSA might contribute to the pathogenesis of TMD through enhancing systemic inflammation. It is therefore not surprising that SA patients have higher TMD incidence. Thirdly, malocclusion (misalignment of the mandible to the cranium) may prevent the airway from staying open during sleep, thus, positional therapy and intraoral protrusion devices are sometimes suggested to treat OSA [29]. However, using mandibular-advancement oral appliances as a treatment modality for OSA may also cause TMD [29,30,31,32,33]. Fourthly, TMD and sleep bruxism may concomitantly present in OSA patients. The bruxism episode index (BEI) positively correlated with AHI in patients with mild-to moderate OSA, whereas patients with severe OSA had lower BEI than those with mild-to-moderate OSA [34]. Fifthly, SA patients, mostly OSA, may have craniofacial configurations and/or muscle dysfunction that predispose them to the development of TMD.

A variety of risk factors for TMD have also been reported in the literature. Traditionally, female sex has been suggested as a risk factor for the development of TMD. For example, Contreras et al. found a higher TMD prevalence in women [35]. Sexual hormones, especially estrogen, might play an important role in the sensitivity of pain, even in the muscles of mastication, and therefore, might contribute to the pathogenesis of TMD [36]. A few studies also showed that the tolerance to pain varied in different phases during the menstrual cycle [36]. In the present study, the TMD prevalence was higher in women than in men in both study arm A and B. The multivariable Cox regression analyses models (Table A1 in the Appendix A) also suggested male sex as an independent protective factor (HR [95% CI] = 0.6 [0.5–0.7] and 0.5 [0.4–0.7] in study arm A and B, respectively). In addition, reduced 25-hydroxyvitamin D level is associated with decreased functional capability of musculoskeletal system [37]. Vitamin D deficiency might contribute to the development of TMD through increasing the parathyroid hormone [38]. Since vitamin D deficiency is more prevalent in women, and is usually associated with poorer musculoskeletal health in community-living people in Taiwan [39], it is not surprising that female sex might be a risk factor for TMD. In a previous study by Contreras [35], peptic ulcer disease has been associated with the presence of painful TMD in the univariate analysis, whereas this association was not shown in the multivariable model. In our current study, we found peptic ulcer disease to be an independent risk factor for incident TMD in SA patients.

Our study had several strengths. Firstly, our study is the largest research discussing the association between SA and TMD thus far. Secondly, a population-based study might minimize the risk of selection bias. Thirdly, this is a cohort study with a very long follow-up time. Fourthly, in contrast to the previous studies with cross-sectional designs, this study demonstrated a temporal trajectory and might therefore provide better evidence regarding the association between SA and TMD. 

This study, however, still had a few limitations. Firstly, the diagnoses based on ICD-9-CM codes might be less reliable than those established with standardized diagnostic criteria in clinical trials. However, besides using SA patients identified with only the codes (i.e., suspected SA cohort) for analyses, we also used SA patients diagnosed with SA after PSG (i.e., probable SA cohort) for another set of diagnosis, which found similar results. Secondly, it is impossible to distinguish OSA and CSA with the ICD-9-CM codes. While OSA patients comprise more than 90% of SA patients, we believe that our findings represent the phenomenon in OSA patients. Thirdly, the information of some potential confounders, such as craniofacial structure, masticatory habits, tooth loss, and body mass index, was not available in the database. Matching the case and controls by age and sex might not eliminate the potential confounders. As SA patients had significantly higher prevalence of comorbidities than the control subjects, resulting from multiple factors, multivariable analyses adjusting for the comorbidities might mitigate the bias introduced by the confounders.

## 5. Conclusions

This large population-based cohort study showed a significantly higher TMD incidence in SA patients.

## Figures and Tables

**Table 1:** Baseline characteristics of the study population.

Variables	Study Arm A			Study Arm B		
	Suspected SA	Control A	*p*-Value	Probable SA	Control B	*p*-Value
N	10,408	104,080		4105	41,050	
Sex, n (%)						
Female	3823 (37%)	38,230 (37%)		894 (22%)	8940 (22%)	
Male	6585 (63%)	65,850 (63%)		3211 (78%)	32,110 (78%)	
Age (year), mean ± SD	47.8 ± 14.9	47.8 ± 14.9		47.6 ± 13.3	47.6 ± 13.3	
Age (year), n (%)						
≤40	3531 (34%)	35,310 (34%)		1298 (32%)	12,980 (32%)	
40 < age ≤ 50	2574 (25%)	25,740 (25%)		1139 (28%)	11,390 (28%)	
>50	4303 (41%)	43,030 (41%)		1668 (41%)	16,680 (41%)	
Residency			<0.0001			<0.0001
Northern Taiwan	5605 (54%)	52,331 (50%)		2330 (57%)	20,771 (51%)	
Other areas	4803 (46%)	51,749 (50%)		1775 (43%)	20,279 (49%)	
Monthly income (NT$), median (IQR)	21,900 (1249–42,000)	21,900 (1249–36,300)	<0.0001	26,400 (1249–43,900)	21,900 (1249–42,000)	<0.0001
Monthly income (NT$), n (%)			<0.0001			<0.0001
≤24000	6031 (58%)	65,840 (63%)		2022 (49%)	24,501 (60%)	
>24000	4377 (42%)	38,240 (37%)		2083 (51%)	16,549 (40%)	
CCI score, mean ± SD	1.7 ± 2.1	1.1 ± 1.7	<0.0001	1.8 ± 2	1 ± 1.6	<0.0001
CCI score, n (%)			<0.0001			<0.0001
=0	3419 (33%)	54,800 (53%)		1287 (31%)	21,585 (53%)	
=1	2642 (25%)	23,116 (22%)		1039 (25%)	9407 (23%)	
≥2	4347 (42%)	26,164 (25%)		1779 (43%)	10,058 (25%)	
Underlying diseases, n (%)						
Heart disease	599 (6%)	3030 (3%)	<0.0001	238 (6%)	992 (2%)	<0.0001
Myocardial infarction	157 (2%)	819 (1%)	<0.0001	69 (2%)	314 (1%)	<0.0001
Congestive heart failure	486 (5%)	2467 (2%)	<0.0001	190 (5%)	768 (2%)	<0.0001
Peripheral vascular disease	204 (2%)	1386 (1%)	<0.0001	71 (2%)	523 (1%)	0.0146
Major neurological disorder	1306 (13%)	7425 (7%)	<0.0001	547 (13%)	2530 (6%)	<0.0001
Cerebral Vascular disease	1230 (12%)	6954 (7%)	<0.0001	522 (13%)	2395 (6%)	<0.0001
Dementia	136 (1%)	870 (1%)	<0.0001	49 (1%)	217 (1%)	<0.0001
Hemiplegia	111 (1%)	891 (1%)	0.028	40 (1%)	312 (1%)	0.1365
Chronic pulmonary disease	3427 (33%)	20,451 (20%)	<0.0001	1407 (34%)	7764 (19%)	<0.0001
Connective tissue disease	360 (3%)	1954 (2%)	<0.0001	131 (3%)	665 (2%)	<0.0001
Peptic ulcer disease	3633 (35%)	23,157 (22%)	<0.0001	1431 (35%)	8976 (22%)	<0.0001
Liver disease	2715 (26%)	16,206 (16%)	<0.0001	1188 (29%)	6786 (17%)	<0.0001
Diabetes mellitus	1544 (15%)	10,849 (10%)	<0.0001	649 (16%)	4212 (10%)	<0.0001
Renal disease	593 (6%)	3339 (3%)	<0.0001	238 (6%)	1266 (3%)	<0.0001
Cancer	668 (6%)	4119 (4%)	<0.0001	236 (6%)	1534 (4%)	<0.0001

Abbreviations: SA = sleep apnea; NT$ = New Taiwan Dollar; CCI = Charlson Comorbidity Index; IQR = interquartile range.

**Table 2:** Incidence rate of temporomandibular disorder (TMD) after the index date in each group.

Variables	Study Arm A										Study Arm B									
	Suspected SA				Control A				Crude IRR [95% CI]	Adjusted IRR [95% CI]	Probable SA				Control B				Crude IRR [95% CI]	Adjusted IRR [95% CI]
	N	TMD	PY	IR	N	TMD	PY	IR			N	TMD	PY	IR	N	TMD	PY	IR		
Whole study population	10,408	184	62,825.7	2.9	104,080	715	634,918.6	1.1	2.3 [2.2–2.5] ***	2.0 [1.9–2.1] ***	4105	66	23,204.8	2.8	41,050	239	232,345.4	1.0	3.1 [2.9–3.4] ***	2.5 [2.3–2.7] ***
Stratified analyses																				
Sex																				
Female	3823	88	23,241.7	3.8	38,230	363	236,432.3	1.5	1.7 [1.5–1.9] ***	1.4 [1.3–1.6] ***	894	16	4716.4	3.4	8940	76	47,813.4	1.6	1.5 [1.3–1.9] ***	1.2 [1.0–1.5] *
Male	6585	96	39,584.0	2.4	65,850	352	398,486.3	0.9	3.1 [2.9–3.3] ***	2.7 [2.6–2.9] ***	3211	50	18,488.5	2.7	32,110	163	184,532.0	0.9	4.1 [3.8–4.5] ***	3.4 [3.1–3.7] ***
Age																				
≤50	6105	100	38,558.2	2.6	61,050	415	389,170.4	1.1	2.3 [2.1–2.4] ***	1.9 [1.8–2.1] ***	2437	37	14,314.6	2.6	24,370	130	143,405.1	0.9	2.5 [2.3–2.8] ***	2.5 [2.2–2.8] ***
>50	4303	84	24,267.6	3.5	43,030	300	245,748.2	1.2	2.4 [2.3–2.7] ***	2.1 [2.0–2.3] ***	1668	29	8890.3	3.3	16,680	109	88,940.4	1.2	3.7 [3.3–4.1] ***	2.6 [2.3–3.0] ***
Residents in																				
Northern Taiwan	5605	103	33,394.5	3.1	52,331	377	317,999.4	1.2	2.3 [2.1–2.5] ***	2.0 [1.8–2.1] ***	2330	40	13,652.2	2.9	20,771	135	116,551.1	1.2	3.1 [2.8–3.5] ***	2.7 [2.4–3.0] ***
Other areas	4803	81	29,431.2	2.8	51,749	338	316,919.2	1.1	2.4 [2.2–2.6] ***	2.1 [1.9–2.2] ***	1775	26	9552.7	2.7	20,279	104	115,794.3	0.9	3.0 [2.6–3.4] ***	2.3 [2.0–2.6] ***
Monthly income																				
≤NT$24,000	6031	101	36,318.0	2.8	65,840	424	398,259.6	1.1	2.5 [2.3–2.6] ***	2.1 [2.0–2.3] ***	2022	32	111,75.7	2.9	24,501	127	135,874.1	0.9	3.8 [3.4–4.2] ***	2.7 [2.4–3.0] ***
NT$24,000	4377	83	26,507.8	3.1	38,240	291	236,659.0	1.2	2.2 [2.0–2.4] ***	1.9 [1.8–2.1] ***	2083	34	12,029.2	2.8	16,549	112	96,471.3	1.2	2.5 [2.2–2.8] ***	2.3 [2.1–2.6] ***
Comorbidity																				
No (CCI score = 0)	3419	53	23,465.9	2.3	54,800	307	364,963.6	0.8	2.5 [2.3–2.7] ***	2.4 [2.2–2.7] ***	1287	20	8271.1	2.4	21,585	103	132,653.4	0.8	3.1 [2.7–3.5] ***	2.7 [2.4–3.1] ***
Yes (CCI score ≥1)	6989	131	39,359.8	3.3	49,280	408	269,955.0	1.5	2.0 [1.8–2.1] ***	1.9 [1.7–2.0] ***	2818	46	14,933.7	3.1	19,465	136	99,692.1	1.4	2.6 [2.4–2.9] ***	2.4 [2.2–2.7] ***

The adjusted IRRs were calculated by multivariable analyses adjusting for sex, age, residency, income level, and the presence of various comorbidities (except for the stratifying variable). * *p* < 0.05; *** *p* < 0.0001. Abbreviations: SA = sleep apnea; N = number of patients; TMD = number of patients with temporomandibular disorder; PY = total patient-years; IR = incident rate (incidence of temporomandibular disorder per thousand-patient-years); IRR = incidence rate ratio; CI = confidence interval; CCI = Charlson Comorbidity Index.

**Table 3:** Multivariable Cox regression analyses of the factors contributing to temporomandibular disorder (TMD) in SA patients.

Variables	Suspected SA Patients				Probable SA Patients			
	Maximal Model		Reduced Model *		Maximal Model		Reduced Model *	
	HR [95% CI]	*p*-Value	HR [95% CI]	*p*-Value	HR [95% CI]	*p*-Value	HR [95% CI]	*p*-Value
Sex (male vs. female)	1.0 [0.6–1.5]	0.9027			1.4 [0.6–3.3]	0.4233		
Age > 50 (vs. Age ≤ 50)	1.0 [0.6–1.7]	0.8622			1.8 [0.8–3.8]	0.1289		
Residency in northern Taiwan (vs. in other areas)	1.0 [0.7–1.6]	0.9741			1.3 [0.6–2.6]	0.4782		
Higher income (>NT$24,000) (vs. lower income)	1.0 [0.6–1.5]	0.9131			0.9 [0.5–1.8]	0.8036		
Presence of underlying diseases (vs. absence of the diseases):								
Heart disease	†				†			
Peripheral vascular disease	2.1 [0.7–6.9]	0.2112			2.6 [0.6–11.5]	0.1924		
Major neurological disorder	0.6 [0.3–1.4]	0.2387			0.8 [0.3–2.2]	0.7341		
Chronic pulmonary disease	1.0 [0.6–1.7]	0.8444			0.7 [0.3–1.5]	0.3687		
Connective tissue disease	2.1 [0.9–4.9]	0.0948			2.3 [0.7–8.0]	0.1742		
Peptic ulcer disease	2.0 [1.2–3.1]	0.0056	2.0 [1.3–3.2]	0.0017	2.7 [1.3–5.6]	0.0081	3.1 [1.6–6.1]	0.0010
Liver disease	1.4 [0.8–2.2]	0.2191			1.2 [0.6–2.4]	0.6526		
Diabetes mellitus	1.4 [0.8–2.6]	0.2388			1.7 [0.7–3.8]	0.2247		
Renal disease	0.3 [0.1–1.4]	0.1267			†			
Cancer	2.5 [1.3–4.7]	0.0056	2.4 [1.3–4.4]	0.0065	2.0 [0.7–5.5]	0.1790		

* Multivariable Cox regression model was built by a backward variable selection method, eliminating variables with the *p* value > 0.05. † Hazard ratio cannot be estimated because of small sample size. Abbreviations: SA = sleep apnea; HR = hazard ratio; CI = confidence interval.

## Abbreviations

CCI	Charlson Comorbidity Index
CI	confidence interval
CSA	central sleep apnea
HR	hazard ratio
IR	incident rate
IRR	incidence rate ratio
NHI	National Health Insurance
OSA	obstructive sleep apnea
PSG	polysomnography
SA	sleep apnea
TMD	temporomandibular disorders

## Appendix A

**Table A1:** Multivariable Cox analysis of the contributing factors for the development of temporomandibular disorder.

Variables	Study Arm A (Suspected SA vs. Control A)		Study Arm B (Probable SA vs. Control B)	
	HR [95% CI]	*p*-Value	HR [95% CI]	*p*-Value
SA patients vs. Control subjects	2.0 [1.6–2.6]	<0.0001	2.5 [1.7–3.7]	<0.0001
Male vs. female	0.6 [0.5–0.7]	<0.0001	0.5 [0.4–0.7]	0.0003
Age > 50 vs. Age ≤ 50	0.9 [0.8–1.2]	0.6055	1.3 [0.9–1.8]	0.1564
Residency (Northern Taiwan vs. Other areas)	1.0 [0.9–1.3]	0.6775	1.2 [0.9–1.7]	0.2368
Higher income (>NT$24,000) vs. lower income (≤NT$24,000)	1.2 [1.0–1.4]	0.1276	1.4 [1.0–1.9]	0.0611
Presence of underlying diseases:				
Heart disease	0.6 [0.3–1.1]	0.0840	0.8 [0.3–2.1]	0.6785
Peripheral vascular disease	1.3 [0.6–2.6]	0.5297	2.4 [1.0–5.5]	0.0448
Major neurological disorder	1.1 [0.8–1.5]	0.6464	1.4 [0.9–2.4]	0.1701
Chronic pulmonary disease	1.2 [0.9–1.5]	0.1304	1.1 [0.7–1.6]	0.7958
Connective tissue disease	1.6 [1.0–2.6]	0.0609	1.8 [0.8–3.8]	0.1538
Peptic ulcer disease	1.7 [1.4–2.1]	<0.0001	1.7 [1.2–2.5]	0.0032
Liver disease	1.4 [1.1–1.7]	0.0084	1.2 [0.8–1.7]	0.4586
Diabetes mellitus	1.1 [0.8–1.5]	0.5360	1.2 [0.7–1.9]	0.4809
Renal disease	0.3 [0.1–0.7]	0.0060	0.4 [0.1–1.2]	0.1035
Cancer	2.0 [1.4–2.8]	0.0001	2.3 [1.3–3.9]	0.0031

Abbreviations: SA = sleep apnea; HR = hazard ratio; CI = confidence interval.
